# Methodological Approach to Predicting Lower Limb Blood Flow Restriction Pressure Using Anthropometry and Hemodynamics

**DOI:** 10.3390/life15081267

**Published:** 2025-08-11

**Authors:** Onur Mutlu Yaşar, Veli Volkan Gürses, Ali Erdem Ciğerci, Özkan Güler, Murat Turğut, Oğuz Gürkan, Mustafa Baş, Serhat Özdenk, Fatma Neşe Şahin, Levent Ceylan, Hamza Küçük

**Affiliations:** 1 Faculty of Health Sciences, Izmir Demokrasi University, Izmir 35140, Türkiye; onurmutluyasar@gmail.com; 2 Faculty of Sport Sciences, Bandırma University, Balıkesir 10200, Türkiye; volkangurses@gmail.com; 3 Faculty of Sports Sciences, Kastamonu University, Kastamonu 37100, Türkiye; alierdemcigerci@gmail.com; 4 Faculty of Sports Sciences, Ankara University, Ankara 06830, Türkiye; oguler@ankara.edu.tr (Ö.G.); nesesahin@ankara.edu.tr (F.N.Ş.); 5 Faculty of Sport Sciences, Sinop University, Sinop 57000, Türkiye; mturgut@sinop.edu.tr (M.T.); sozdenk@sinop.edu.tr (S.Ö.); 6 Faculty of Sport Sciences, Yozgat University, Yozgat 66000, Türkiye; oguz.gurkan@bozok.edu.tr; 7 Faculty of Life Sciences, University of Health Sciences, Istanbul 34744, Türkiye; mustafa.bas@sbu.edu.tr; 8 Faculty of Sport Sciences, Hitit University, Corum 19030, Türkiye; leventceylan17@hotmail.com; 9 Yasar Dogu Faculty of Sport Sciences, Ondokuz Mayis University, Samsun 55200, Türkiye

**Keywords:** BFR, muscle function, anthropometry, hemodynamics, exercise

## Abstract

Blood flow restriction (BFR) training, first introduced by Dr. Yoshiaki Sato as KAATSU, has attracted increasing interest in sports science. Although the BFR training method has several additional benefits, the way occlusion pressure is identified contributes to BFR usability and safety. This study aims to develop and validate a predictive model for estimating individualized LOP with manual technique by examining the influence of blood pressure, fat percentage, limb circumference, and BMI as independent variables. A total of 158 healthy male adults participated in the study. Subjects with cardiovascular, metabolic, or musculoskeletal disorders, as well as those using supplements, medications, or nicotine, were excluded from the study. The cross-sectional study examined the correlation between the variables and derived a regression equation for predicting the corresponding LOP and anthropometric values. Three measurements were taken and the arithmetic mean was calculated from these measurements. It is evident that body mass index (BMI) emerged as a significant contributing factor in predicting limb occlusion pressure (LOP), outpacing traditional anthropometric variables such as limb circumference or body fat percentage. The regression model accounted for 18% of the variance in LOP (R^2^ = 0.18), with a standard error of estimate (SEE) of 20.5 mmHg, suggesting moderate predictive capacity. Future work should investigate vascular factors and technological development in BFR devices for optimal pressure calibration to improve the efficacy of individualized BFR training.

## 1. Introduction

Blood flow restriction (BFR) training, originally developed by Dr. Yoshiaki Sato as the KAATSU strength training method, has gained attention in sports science. Studies show that shorter training sessions with increased volume can lead to muscle hypertrophy, even with lighter loads than traditional resistance training [1,2,3,4,5,6,7]. The technique reduces joint forces and improves recovery, muscle hypertrophy, and adaptation, and is likely favorable for people who cannot move heavier loads due to a high risk of injury or other physical issues [8,9,10,11,12]. Under BFR, venous blood flow is limited, but arterial blood flow persists, resulting in a physiological state that induces metabolic and endocrine signaling, such as lactate production, growth hormone release, and intramuscular pathways relevant to hypertrophy [3,13,14,15,16]. Although the BFR training method has several additional benefits, the way occlusion pressure is identified contributes to BFR usability and safety [17,18,19,20,21,22].

Hypoxia also affects the compliance of small- and large-caliber arteries and arterioles in a different pattern, resulting in additional vasodilatory recruitment. During hypoxia, slow-twitch (Type I) fibers become fatigued early [23]. Thus, in conditions where the normal system requires a load to engage more Type II fibers, adjustments are made to facilitate movement. The increase in hypertrophy and strength with the BFR method is explained by this mechanism. BFR induces favorable vascular adaptations, such as increased capillary density and endothelial function, that may improve muscle endurance and cardiovascular health over time [24,25,26,27,28]. This is a systemic adaptation, not a muscular effect, meaning it can also be beneficial to populations with cardiovascular restrictions. The evidence noted that BFR can be a promising tool for many of these populations, including postoperative patients, the elderly, and those undergoing musculoskeletal rehabilitation [29,30,31,32]. Several studies have demonstrated that BFR rehabilitation programs can provide significant benefits for postoperative patients, elderly groups, and people with musculoskeletal pathology [8,33,34,35,36]. Patients recovering from anterior cruciate ligament (ACL) reconstruction have shown reduced muscle atrophy and improved strength gains when BFR was incorporated into their rehabilitation protocols [37,38,39,40]. In addition, BFR is considered a potential method for people with sarcopenia, osteoporosis, and cardiovascular disease, as it provides an alternative to traditional resistance training while minimizing joint stress [14,41,42,43]. However, concerns about long-term safety, including potential risks such as deep vein thrombosis (DVT), nerve compression, and excessive muscle damage, emphasize the need for continued research to develop BFR applications.

This mechanism establishes a distinct physiological setting that facilitates metabolic and endocrine adjustments [44,45,46,47,48]. Despite evidence showing that BFR induces adaptations comparable to those from high-load resistance training [49,50,51], differences in populations and protocols are among the variations attributable to individual characteristics such as limb circumference, muscle makeup, vascular reactivity, and experience. The main controversy is whether fixed pressure or individualized occlusion pressure will yield the best outcomes [52,53,54]. Selecting an incorrect pressure may cause inefficient adaptation, excessive discomfort, or increased vascular risks. Moreover, the BFR method has been primarily studied in resistance training, but the practice remains controversial in endurance training. Whereas some studies demonstrate that low-intensity aerobic training combined with BFR can cause cardiovascular adaptations and hypertrophy, other studies report opposite results [55,56,57]. The differences may depend on occlusion duration and intensity.

The accurate calculation of limb occlusion pressure (LOP) is a fundamental aspect of BFR training that is necessary for the safety and effectiveness of the process [58,59]. Usually, LOP is measured with a pneumatic tourniquet, while Doppler ultrasound is regarded as the gold standard for accuracy [60]. Nevertheless, there are additional options, including portable Doppler systems and specific BFR cuffs, that are more affordable and more convenient to use, but they are not precise enough for such measurements [61]. If sophisticated equipment is absent, manual approaches may be applied, though they are unlikely to be accurate [62]. The choice of measurement method has a great effect on the results of BFR. Therefore, it is necessary to implement the individual approach based on limb circumference, blood pressure, and cuff properties [63,64].

Currently, there is no consensus on which criteria should be used to estimate optimal occlusion pressure, and a significant number of the existing guidelines do not personalize the pressure values. Given the wide range of responses that can be expected depending on anthropometric and physiological characteristics, there is a need for a standardized yet individualized estimation of LOP. The impact of systolic and diastolic blood pressure, limb circumference, BMI, fat percentage, and lean mass has been examined in only a few studies as the factors that may potentially determine optimal occlusion pressure. This study hypothesizes that a predictive model incorporating physiological (blood pressure, fat percentage, and lean mass) and anthropometric (limb circumference and BMI) factors can accurately estimate individualized LOP, leading to improved safety and efficacy in BFR training. Furthermore, future studies are needed to determine physiological adaptations compared to fixed pressure methods, minimizing vascular risks while optimizing muscle hypertrophy and performance outcomes.

This study aims to examine the associations between physiological and anthropometric variables and LOP estimation through a regression model for estimating individualized LOP with manual technique by examining the influence of blood pressure, fat percentage, limb circumference and BMI independent variables. By integrating physiological and anthropometric factors, we seek to establish a more precise and individualized approach to BFR prescription. The findings of this research could enhance both performance-based applications and clinical rehabilitation protocols, contributing to safer and more effective BFR training methodologies. The study further aims to compare the effectiveness of individualized LOP estimation with fixed occlusion pressures.

## 2. Materials and Methods

### 2.1. Participants

A total of 158 healthy male adults participated in the study. Participants were recruited from a university population through voluntary participation. Inclusion criteria required participants to be healthy males aged 18–35 years, non-smokers, not currently using any medications or dietary supplements, and engaging in regular physical activity. Exclusion criteria included the presence of known cardiovascular, metabolic, or musculoskeletal disorders. To determine eligibility, participants completed a standardized health screening questionnaire that included items related to cardiovascular symptoms (e.g., chest pain, shortness of breath, dizziness during exertion) and medical history (e.g., hypertension, arrhythmia, myocardial infarction, or family history of heart disease). In cases of uncertainty, participants were interviewed individually by a trained researcher to verify their self-reported data. Participants who reported any relevant symptoms or conditions were excluded from the study. All subjects participated in the study in a single testing session in the laboratory. They were evaluated 2 h after their meal and asked not to consume caffeine or exercise on the day of the test. The study procedures followed the Declaration of Helsinki and were approved by the Institutional Review Board at the Bandirma Onyedi Eylul University (2024-885), and written informed consent was obtained from all subjects prior to their participation.

### 2.2. Study Design

This study aimed to establish a regression model for limb occlusion pressure using anthropometric and hemodynamic variables in healthy male participants. The cross-sectional study examined the correlation between the variables and derived a regression equation for predicting the corresponding LOP and anthropometric values. The study aimed to derive a predictive equation for LOP based on independent variables: systolic blood pressure (SBP), diastolic blood pressure (DBP), thigh circumference (TC), body mass index (BMI), and body fat percentage (BFP, %), as these factors may affect the optimal arterial occlusion pressure required for blood flow restriction training. To establish a reference standard for LOP, a medical-grade pneumatic tourniquet (Reister Pneumatic Cuff; Rudolf Riester GmbH, Jungingen, Baden-Württemberg, Germany) was used. Participants were instructed to rest in a seated position for 10 min in a quiet room prior to measurements to minimize acute hemodynamic fluctuations. A pneumatic cuff (13 cm width, 96 cm length) was wrapped around the proximal thigh, just below the inguinal crease, ensuring uniform compression. The cuff was gradually inflated until arterial pulse occlusion was confirmed via Doppler ultrasound (Bidop 3, Hand-held Vascular Doppler; Hadeco, Inc., Kawasaki City, Kanagawa, Japan). This measure served as the gold standard for determining LOP in each subject. All measurements were conducted by the same trained examiner to minimize inter-observer variability. To ensure consistency, standardized measurement protocols were strictly followed. Prior to collecting data, subjects were screened for eligibility based on predetermined criteria for inclusion and exclusion. All subjects were asked to remain seated and to rest for 10 min before measures were taken to allow their hemodynamics to stabilize. A 10 min delay was maintained between measures to eliminate any potential carryover. The legs of the participants were measured separately to compensate for the possibility of different occlusion pressures. Research seems to indicate that increased limb circumference also correlates with increased occlusion pressure [60], indicating the need to test the model in a variety of limb segments. To reduce inter-rater effects, the same trained experimenter conducted all measures for the arms, and a different trained experimenter conducted all measures for the legs. A total of 77 subjects were recruited for this study, with an additional 81 subjects completing the full validation procedure on the legs, making a total of 158 subjects completing the full validation procedure on the legs. Participants were instructed to refrain from strenuous exercise and caffeine consumption for at least 24 h before testing. All assessments took place in a temperature-controlled environment (23 °C ± 1 °C) to reduce external variability. By integrating physiological and anthropometric markers, this study aims to establish an individualized approach to pressure prescription and a practical approach for estimating LOP, contributing to the safer and more effective application of blood flow restriction training.

### 2.3. Procedures

#### 2.3.1. Anthropometric Measurements

Anthropometry measurements followed proper guidelines to achieve both accuracy and precision. The anthropometric measurements—body mass (BM, kg) and height (cm)—were taken with a sufficiently precise digital scale (TANITA BC-558 IRONMAN®, Segmental Body Composition Monitor; Tanita Corporation, Tokyo, Japan; 0.05 kg precision) and a stadiometer (Seca 213 Stadiometer; Seca GmbH & Co. KG, Hamburg, Germany; 0.1 cm precision). At the time of measurements, subjects did not wear any footwear and had minimal clothing (underwear). Proximal TC was measured on the gluteal fold level using an anthropometric tape (Seca 201 Ergonomic Circumference Measuring Tape; Seca GmbH & Co. KG, Hamburg, Germany). There were three measurements, and the final TC value was calculated by using the arithmetic mean function. To maintain optimal conditions for measurement, subjects fasted for at least 8 h before the assessment, drank no caffeinated drinks for 24 h, and emptied their bladder a few minutes before the evaluation took place. Also, they removed all metallic items, such as earrings, rings, and glasses, since those can interfere with the impedance and wore only standardized clothing.

#### 2.3.2. Hemodynamic Measurements

After a ten-minute sitting rest allowing for hemodynamic stabilization, brachial blood pressure was measured with a standardized automatic oscillometric instrument (Omron M2 HEM-721, Omron Healthcare, Tokyo, Japan). The assessments took place on the left arm. Three measurements were taken 1 min apart. To improve the reliability of the measurement, the systolic blood pressure (SBP, mmHg) and diastolic blood pressure (DBP, mmHg) were calculated as the mean of the last two values.

#### 2.3.3. Determination of the Arterial Occlusion Pressure

##### Determination of Arterial Occlusion Pressure (AOP)

The arterial occlusion pressure (AOP) was measured using a standardized Doppler ultrasound procedure. Participants were positioned supine on an examination table, and a pneumatic cuff (13 cm width, 96 cm length; Riester Pneumatic Cuff, Rudolf Riester GmbH, Jungingen, Baden-Württemberg, Germany) was placed around the proximal thigh, immediately distal to the inguinal fold. A handheld Doppler ultrasound device (Bidop 3, Hand-held Vascular Doppler; Hadeco, Inc., Kawasaki City, Kanagawa, Japan) with an 8 MHz probe was used to detect arterial pulse. The Doppler ultrasound probe was placed at approximately a 45-degree angle over the posterior tibial artery, posterior to the medial malleolus. The cuff pressure gradually increased in increments of 40 mmHg until the Doppler ultrasound signal indicated complete cessation of arterial pulse, defining the arterial occlusion point. Once pulse occlusion was confirmed, the cuff pressure slowly decreased in 10 mmHg intervals until arterial pulse was audibly restored, confirming the accuracy of occlusion pressure. Three measurements were taken with a minimum of 10 min rest periods between each measurement to avoid hemodynamic carry-over effects, and the meaning of these measurements was used as the individual’s AOP. The LOP was documented as “+300 mmHg” if occlusion was not achieved at 300 mmHg. Outlier regression analysis was conducted to exclude participants in whom occlusion was not established.

#### 2.3.4. Predictor Variables

The selection of predictor variables for the regression model was guided by previous literature identifying physiological and anthropometric factors that influence limb occlusion pressure (LOP). Specifically, systolic blood pressure (SBP) and diastolic blood pressure (DBP) were included due to their well-documented associations with arterial stiffness and vascular resistance, which affect the pressure required to achieve occlusion during BFR application [1,2]. Anthropometric variables such as thigh circumference (TC) and body mass index (BMI) have been previously correlated with limb size, soft tissue thickness, and external pressure distribution, all of which influence occlusion thresholds [3,4,5]. Body fat percentage (BFP) was selected to further account for interindividual differences in tissue composition, which may modify the transmission and absorption of cuff pressure [6]. Together, these variables were chosen to reflect relevant structural and hemodynamic characteristics, enabling a more individualized and accessible estimation of LOP without the need for Doppler ultrasound or specialized equipment.

#### 2.3.5. Intra-Rater Reliability

To assess intra-rater reliability of arterial occlusion pressure (AOP) measurements, the same trained examiner performed repeated measurements on a subgroup of 20 participants under identical experimental conditions. Intraclass correlation coefficient (ICC) with a two-way mixed-effects model for absolute agreement was calculated.

### 2.4. Statistical Analysis

The data were analyzed using SPSS version 27.0 (SPSS Inc., Chicago, IL, USA). Data are expressed as means ± standard deviations. The threshold for statistical significance was established at *p* < 0.05. Standard deviations (SD) were used to express all variations within the data.

Normality of the data distribution was verified using the Shapiro–Wilk test. Pearson’s correlation coefficients (r) were calculated to assess the strength of associations between anthropometric and hemodynamic variables identified as potential predictors of LOP. The primary objective of this analysis was to evaluate the strength of associations between anthropometric and hemodynamic variables that had been identified as potential predictors of LOP. The secondary objective entailed the identification of multicollinearity among independent variables associated with LOP. To evaluate which variables predicted the limb occlusion pressure at which arterial occlusion occurred, three different hierarchical linear regression models with sequential blocks were used for the limb. Power analysis, carried out using G*Power 3.1.9.2, indicated that a minimum of 36 participants was required to fit a regression equation for LOP, considering 3 predictor variables, with an effect size (f^2^) of 0.35, statistical power of 0.80, and a two-tailed significance level (α) of 0.05. To increase the power of the predictive model to achieve an effect size of 0.15, which required a minimum of 77 participants; 158 participants were selected. The first block included variables that are easily measured by individuals: systolic blood pressure (SBP), diastolic blood pressure (DBP), and thigh circumference (TC), as these were hypothesized to be the primary predictors of LOP. Subsequent blocks incorporated the body mass index (BMI) and body fat percentage based on their presumed influence on AOP. To have confidence in the results of hierarchical multiple regression, in the final adjusted model, variables were retained only if they had a *p*-value less than 0.30 in the initial or preliminary input model. An evaluation was conducted for each regression block to determine changes in Pearson correlation, PCC, R^2^, SEE, and F-value. Collinearity diagnostics involved calculating the variance inflation factor (VIF) and tolerance (T) values, with criteria VIF < 5 and T > 0.1 to ensure model stability. Intra-rater reliability for arterial occlusion pressure (AOP) measurements was evaluated using an intraclass correlation coefficient (ICC) based on a two-way mixed-effects model with absolute agreement. ICC values greater than 0.75 were considered indicative of good reliability.

## 3. Results

Table 1 presents the demographic characteristics of the participants (*n* = 158). Table 2 presents the data on demographic characteristics. Table 2 presents Pearson’s correlation coefficient values corresponding to the assessed variables. Positive relationships were identified between selected predictor variables (SBP, DBP, TC, BMI, and BFP) and the predicted variables. All were associated with LOP (*p* < 0.05). As illustrated in Table 2, SBP and BMI demonstrated moderate correlation coefficients (r = 0.309 and 0.302, respectively), indicating a moderate relationship with LOP. As demonstrated in Table 2, the Pearson’s correlation coefficient values corresponding to the assessed variables reveal that some of the predictor variables (SBP, DP, TC, BMI, and BFP) exhibited positive relationships with the predicted variables. Furthermore, the findings indicate that all variables were associated with LOP (*p* < 0.05). Specifically, SBP and BMI demonstrated high correlation coefficient values (r = 0.309 and 0.302, respectively), denoting a moderate relationship with LOP. In addition, DP, TC, and BFP exhibited weaker relationships with LOP (r = 0.228, 0.130, and 0.149, respectively).

Table 3 presents the results of hierarchical regression models to predict the LOP, which were obtained from statistical calculations. The model aimed to predict LOP using handled Doppler measurements. The intra-rater reliability for arterial occlusion pressure (AOP) measurements indicated excellent consistency, with an intraclass correlation coefficient (ICC) of 0.91 (95% CI: 0.86–0.95). Block 1 included SBP, DBP, and limb circumference and accounted for a significant portion of the variance (R^2^ = 113, SEE = 21.527, F = 6.586, *p* < 0.000). Standardized beta values and partial correlation coefficients indicated that DB was the most influential predictor (β = 5.106, part = 0.227). SBP, DBP, and TC explained 9.6% of the variation in AOP. Block 2 included SBP, DBP, limb circumference, and BMI; these accounted for a significant portion of the variance (R^2^ = 188, SEE = 20.639, F = 8.930, *p* < 0.000). Standardized beta values and partial correlation coefficients indicated that SB was the most influential predictor (β = 4.806, part.060). SBP, DBP, TC and BMI explained 17% of the variation in AOP. Block 3 included SBP, DBP, and limb circumference; BMI and percentage of body fat accounted for a significant portion of the variance (R^2^ = 175, SEE = 20.539, F = 7.714, *p* < 0.000). Standardized beta values and partial correlation coefficients indicated that SB was the most influential predictor (β = 5.371, partial correlation = 0.233). SBP, DBP, TC, BMI and PBF explained 18% of the variation in AOP.

Based on these findings, the regression equations for LOP in the limb for model blocks are as follows:

LOP (mmHg) = 90.326 (5.907 × SBP),
LOP (mmHg) = 65.191 + (4.806 × SBP) + (1.679 × DBP) − (−0.689 × TC) + (2.682 × BMI).

## 4. Discussion

The present study aimed to develop a model for estimating lower limb occlusion pressure (LOP) based on anthropometric and hemodynamic factors. The results show that 9.6% of LOP variance is accounted for by a combination of systolic blood pressure (SBP), diastolic blood pressure (DBP), and thigh circumference (TC). Adding body mass index (BMI) to the combination increased the estimate to 17% (r = 0.75), while adding body fat percentage (PBF) to the model increased it to 18%, with a diminished effect size (r = 0.13). It follows that SBP, DBP, and TC contribute to the estimation of LOP; however, BMI was identified as a significant predictor of the occlusion pressure necessary for blood flow restriction training. The results corroborate previous research into the importance of BMI as a predictor of LOP. For instance, Loenneke et al. (2012) showed that higher BMI individuals required more cuff pressure to cause arterial occlusion, likely due to higher subcutaneous adipose tissue and muscle mass [65]. Jessee et al. (2016) also reported a significant correlation between BMI and LOP, stating that overall body composition affects vascular occlusion thresholds [66]. Lastly, Patterson et al. (2019) found that the limb circumference was a better predictor of LOP than another metric, which is consistent with results of the current study [67]. However, the role of the percentage of body fat in predicting LOP is ambiguous. While some studies [68,69] have found that PBF could be a determinant of LOP, we found that adding PBF as a predictor to the regression model contributed only marginal improvement to the overall predictive power. An explanation could be that the localization of body fat varies significantly among individuals, which changes the compression of tissues and arterial occlusion in unique ways. Indeed, Murray et al. (2021) reported that visceral fat had a greater impact on vascular occlusion compared to subcutaneous fat, suggesting that general measures of PBF are not sufficient to estimate LOP in the model reliably [18].

Contrary to our results, limb circumference is considered a more relevant factor than BMI in estimating LOP in some studies. For instance, Mouser et al. (2017) found that thigh circumference was the strongest predictor of occlusion pressure in both trained and untrained individuals [70]. This finding could be attributed to the differences in measurement techniques, participant population, or BFR devices employed. Unlike our study, which incorporated a multi-predictor, hierarchical regression model, Mouser et al. (2017) primarily used single-variable analyses, potentially overestimating the contribution of limb circumference to LOP [70]. Additionally, Zeng et al. (2019) found that arterial compliance and pulse wave velocity were better predictors of LOP than traditional anthropometric measures [71]. It appears that the vascular characteristics, rather than overall body composition, are critical factors determining the pressure necessary for training using BFR. This could be described as a limitation of the current study, which did not involve direct vascular measurements in the experimental setup.

The second study by Kilgas et al. (2019) suggested that physiological factors like hydration status and muscle perfusion could be major contributors to LOP, regardless of BMI or limb circumference [72]. Since our research did not control such physiological differences, future studies may consider hydration and muscle oxygenation assessments to improve models for predicting LOP. This work reveals that BMI is a better indicator of the level of pressure (LOP) than previously considered anthropometric and hemodynamic variables. Still, the final model has low practicality, which suggests that the consideration of these factors alone may not result in accurate pressure calibration for BFR. With more widespread use of automated BFR devices featuring real-time pressure adjustments, future research should investigate how technological developments may improve LOP estimation [73]. Furthermore, since the evidence concerning this topic is mixed, future work should consider a more integrated approach, to include vascular parameters such as arterial stiffness, endothelial functioning, and regional blood flow dynamics [18]. Based on the longitudinal variability of LOP, further research would also benefit from investigating the ways in which training adaptations and physiological changes influence the mean LOP of BFR practitioners. While the predictive model demonstrated statistically significant associations between selected variables and limb occlusion pressure (LOP), the standard error of estimate (SEE) of approximately 20.5 mmHg represents a considerable degree of variability. From a clinical perspective, such an error margin could result in substantial overestimation or underestimation of LOP in certain individuals, potentially increasing the risk of adverse effects such as excessive discomfort, nerve compression, or insufficient training stimulus. Therefore, although the model may offer a practical estimation method when Doppler ultrasound is unavailable, its application should be approached with caution in high-risk populations or clinical settings. Future refinements are necessary to improve predictive precision and reduce the SEE to clinically acceptable levels, ideally below 10–15 mmHg.

The present study has some drawbacks despite its usefulness. Firstly, the sample drawn from the study comprised individuals at a specific point in time, which could limit the generalizability of the findings. Future research should consider employing a more heterogeneous sample, with participants including people of varying fitness levels, ages, and clinical conditions. Secondly, the study did not include vascular properties such as arterial elasticity or venous return but instead focused on anthropometric and hemodynamic variables. Thirdly, the study employed a cross-sectional design, preventing cause-and-effect conclusions and necessitating longitudinal research. Given the model’s limited explanatory power (R^2^ = 0.18), its practical use should be approached with caution. Although the model is not yet suitable for clinical implementation, it provides a foundation for future efforts to personalize BFR protocols. While this study provides a novel approach for estimating individualized limb occlusion pressure (LOP), it does not include a comparison with traditional fixed-pressure BFR protocols. Future research should directly compare the effectiveness, safety, and physiological outcomes of individualized LOP-based prescriptions versus standardized fixed-pressure approaches. Such comparisons would offer valuable insights into the clinical utility and performance advantages of personalized BFR strategies across diverse populations. Future studies should explore more sophisticated predictive frameworks, such as machine learning algorithms, and incorporate vascular imaging modalities (e.g., Doppler ultrasound, elastography) to enhance the precision and clinical applicability of LOP estimation models.

## 5. Conclusions

In conclusion, this study developed a regression-based model incorporating blood pressure and anthropometric variables to estimate limb occlusion pressure (LOP) in healthy males. While the model demonstrated statistically significant associations, its predictive capacity was modest (R^2^ = 0.18), and the standard error of estimate (SEE ≈ 20.5 mmHg) limits its practical application in clinical settings. Therefore, the proposed model may serve as a preliminary step toward individualized BFR prescription, but further refinement and validation are necessary before routine use. Overall, from our work, it is evident that BMI emerged as a significant contributing factor in predicting LOP, outpacing traditional anthropometric variables such as limb circumference or body fat percentage. The relatively weak predictive power highlights the need to incorporate additional physiological parameters for more accurate estimation. Future research should focus on integrating vascular factors and advancing BFR technologies—such as the use of vascular imaging and machine learning approaches—to improve predictive accuracy and potentially enhance training outcomes in rehabilitation settings.

### Limitations and Suggestions

Only male participants were included in the study. One of the important limitations of this study is the homogeneous sample, which consisted exclusively of healthy adult males. As such, our findings may not be generalizable to female populations, older adults, or individuals with comorbidities. Sex-based and age-related physiological differences—particularly in vascular compliance, limb composition, and hormonal responses—may be associated with training adaptations and the relationship between anthropometric variables and limb occlusion pressure. Future studies should aim to include more diverse populations to improve external validity and better inform personalized BFR protocols across demographics. In other studies, including female participants may be useful for evaluating the gender variable. In addition, the age range of the participants in the study was limited to 18–27 years. In this context, it may be useful to keep the age range wide in future studies. During the application carried out within the scope of the study, a handheld Doppler was used to limit blood pressure. Using digital Doppler in other studies likely to be conducted may support a different perspective. Although blood flow restriction (BFR) training is generally considered safe when applied appropriately, it is not without risks. Potential complications such as deep vein thrombosis (DVT), nerve compression, and localized discomfort have been reported in the literature, particularly when excessive cuff pressures are used or individual vascular characteristics are not considered. While no adverse events occurred in the present study, the risk of such complications underscores the importance of individualized pressure prescription, as well as proper screening and supervision in clinical and athletic settings. Furthermore, this study did not implement internal validation techniques such as k-fold cross-validation or bootstrapping to assess the generalizability of the regression model. As a result, the model’s performance may be overestimated due to potential overfitting. Future studies should incorporate robust validation methods to enhance the reliability and predictive stability of LOP estimation models in independent samples.

## Figures and Tables

**Table 1:** Descriptive characteristics of the subjects (*n* = 159).

Variables	Mean ± SD	Min–Max
Age (years)	22.22 ± 1.81	18–27
Body mass (kg)	75.29 ± 11.34	47.4–105.7
Body fat (%)	18.84 ± 5.88	6.20 ± 33.80
Height (cm)	176.79 ± 6.89	153.20–190.20
BMI (kg/m^2^)	24.06 ± 3.18	16.99–34.51
TC (cm)	54.47 ± 4.85	44.00–68.00
SBP (mmHg)	123.0 ± 12	100–159
DBP (mmHg)	69.6 ± 9.6	50–90
LOP (mmHg)	211.04 ± 32.86	160–255

BMI = body mass index; TC = thigh circumference; SBP = brachial systolic blood pressure; DBP = brachial diastolic blood pressure; LOP = limb arterial occlusion pressure.

**Table 2:** Pearson’s correlation coefficients between predictor and predicted variables (*n* = 158).

		Predictor Variables				
Predicted Variable LOP		SBP (mmHg)	DBP (mmHg)	TC (cm)	BMI (kg/m^2^)	BFP (%)
LOP	r	0.309	0.228	0.130	0.302	0.149
	*p*	0.000	0.002	0.049	0.000	0.031

LOP = limb arterial occlusion pressure; SBP = brachial systolic blood pressure; DBP = brachial diastolic blood pressure; TC = thigh circumference; BMI = body mass index; BFP = body fat percentage.

**Table 3:** Model for the lower body (*n* = 158).

Model Blocks							
Block 1							
	Stand. B	*p*	Part	95% CI Lower	95% CI Upper		
SBP	5.907	<0.001	0.309	1.25	6.04		
	R	R^2^	SEE			Mean Square Eror	Sig F change
	0.95	0.90	21.577			465.578	<0.001
Block 2							
	Stand. B	*p*-value	Part	95% CI Lower	95% CI Upper		
SBP	4.997	0.004	0.233	0.49	5.22		
DBP	2.129	0.310	0.077	−2.88	4.20		
	R	R2	SEE			Mean Square Eror	Sig F change
	0.101	0.090	21.575			465.469	0.310
Block 3							
	Stand. B	*p*-value	Part	95% CI Lower	95% CI Upper		
SBP	5.106	0.003	0.227	0.54	6.21		
DBP	1.678	0.427	0.072	−3.02	3.98		
TC	0.509	0.156	0.108	0.13	1.21		
	R	R^2^	SEE			Mean Square Eror	Sig F change
	0.113	0.096	21.504			462.414	0.156
Block 4							
	Stand. B	*p*-value	Part	95% CI Lower	95% CI Upper		
SBP	4.806	0.004	0.214	0.45	6.13		
DBP	1.679	0.408	0.060	−3.01	3.99		
TC	−0.689	0.156	−0.107	−0.148	1.16		
BMI	2.682	<0.001	0.274	−0.47	1.28		
	R	R2	SEE			Mean Square Eror	Sig F change
	0.188	0.167	20.639			425.951	<0.001
Block 5							
	Stand. B	*p*-value	Part	95% CI Lower	95% CI Upper		
SBP	5.371	0.002	0.233	0.46	6.20		
DBP	1.878	0.353	0.067	−2.99	4.09		
TC	−0.683	0.143	−0.106	−0.146	1.16		
BMI	3.233	<0.001	0.297	−0.48	1.34		
BFP	−0.557	0.116	−0.114	−0.55	0.432		
	R	R^2^	SEE			Mean Square Eror	Sig F change
	0.201	0.175	20.539			421.832	0.116

*p* = *p*-value; Part = partial correlation coefficient; CI = confidence interval; LOP = limb arterial occlusion pressure; SBP = brachial systolic blood pressure; DBP = brachial diastolic blood pressure; TC = thigh circumference; BMI = body mass index; BFP = body fat percentage.

## Data Availability

The data presented in this study are available on request from the corresponding author.
